# Twisting Tube Artificial Muscle (TTAM) and Its Application in Agonist and Antagonist Drive

**DOI:** 10.3390/biomimetics11010038

**Published:** 2026-01-05

**Authors:** Jiutian Xia, Jialong Cao, Tao Ren, Yonghua Chen, Ye Chen, Yunquan Li

**Affiliations:** 1 The Shien-Ming Wu School of Intelligent Engineering, South China University of Technology, Guangzhou 510641, China; 202210190721@mail.scut.edu.cn (J.X.); 202521065005@mail.scut.edu.cn (J.C.); 2 School of Mechanical and Electrical Engineering, Chengdu University of Technology, Chengdu 610500, China; 3 The Department of Mechanical Engineering, The University of Hong Kong, Hong Kong 999077, China

**Keywords:** fluidic artificial muscles, twisting tube actuation, agonist and antagonist drives, soft robotics

## Abstract

Pneumatic artificial muscles (PAMs) are inherently compliant and relatively safe. They are widely used in applications where human beings and robots interact closely, such as service robots or medical robots. However, PAMs are constrained by bulky pumps and valve control systems, limiting their mobility, portability, and practical applications. In this research, a novel type of artificial muscle, namely Twisting Tube Artificial Muscle (TTAM), is presented. In a TTAM design, fluid (pressurized air in this research) is contained inside an elastic tube (constrained by a braiding). By twisting the tube from one end, the fluid inside the twisted part will be extruded to the untwisted part, resulting in a pressure increase inside the untwisted part. Both the twisted and untwisted parts will thus contract. Modeling and experimental characterization of the TTAM are conducted. In an experimental test at 100 kPa initial air pressure, after a 6π twisting angle, the internal pressure of a prototype TTAM is increased to 219 kPa, and the largest contraction force of the TTAM was up to 200 N. A novel antagonistic robotic joint actuated by two TTAMs is developed as a sample application.

## 1. Introduction

Robots are increasingly designed to operate in close physical proximity to humans; consequently, the likelihood of unintended collisions with humans or the environment has increased substantially [1]. Although robust sensors and precise control systems enable collision detection [2] and active compliance control [3], sensor failures, low sampling rates, or limited system bandwidth can compromise the safety of humans, the environment, and even the robot itself [2,4].

Developing robots with inherent compliance can markedly mitigate these risks. A feasible strategy for compliant robotics is to develop bioinspired artificial muscles modeled on the highly flexible, naturally compliant musculature of organisms such as mollusks and mammals [5,6]. Accordingly, a wide range of artificial muscles has been proposed based on diverse actuation mechanisms, including soft fluidic actuators (SFAs) [7], electroactive actuators (e.g., dielectric elastomer actuators, DEAs) [8], and thermally activated actuators (e.g., shape memory alloys, SMAs [9]; shape memory polymers, SMPs [10]; and coiled polymer composite fibers [11,12]). Among these, SFAs are arguably the most widely adopted in both academic and industrial settings owing to their safety, low cost, ease of fabrication, and ability to deliver large deformations and high force output [7]. SFAs encompass multiple configurations, one of which is the pneumatic artificial muscle (PAM). PAMs have been studied and deployed for decades, with early developments dating back to the 1950s [13,14]. A typical PAM consists of an inflatable elastomeric tube (e.g., latex or silicone rubber) constrained by a braided mesh sleeve made of nylon fibers [15,16]. PAMs offer a high load-to-weight ratio, tunable stiffness with spring-like behavior, and nonlinear passive elasticity [17]. These properties have enabled their use in compliant robotic arms [18,19] and in medical devices such as wearable rehabilitation systems [20,21].

Despite these advantages, conventional PAMs (and, more broadly, SFAs) face challenges that restrict their widespread deployment. They typically require tethered pneumatic infrastructure (e.g., pumps or compressors, valves, and regulators), which increases system bulk and mass and constrains portability and usable workspace. Moreover, accurate control is often challenging due to gas compressibility, flow dynamics, and structural nonlinearities in soft materials, particularly when the available control bandwidth is limited [18,19,20,21]. Collectively, these limitations motivate the development of untethered, compact, and readily controllable compliant actuation technologies.

To reduce tethering and improve practicality, recent studies have explored self-contained or untethered fluid-driven actuation [22,23]. Sealing a prescribed amount of working fluid (pressurized gas or liquid) within an SFA and regulating its internal distribution via external stimuli is an effective strategy for achieving untethered and readily controllable fluidic actuation. For example, electrohydraulic actuators redistribute a sealed liquid under electrical inputs to generate muscle-like contraction. HASEL-type actuators exemplify this approach, enabling electrically addressable, high-performance soft actuation without external pneumatic supplies [24]. Mirvakili et al. [13] proposed an untethered PAM design that utilizes electromagnetic induction to heat a pre-sealed liquid containing magnetic particles. The resulting liquid-to-gas phase transition increases the internal pressure and thereby produces muscle contraction. Other designs generate the required external stimuli using motors. For example, Usevitch [25] designed an untethered isoperimetric soft robot consisting of a soft robotic truss formed from thin-walled inflatable tubes. Actuation is achieved by driving a roller module through the tubes to redistribute the gas, thereby changing the robot’s shape; importantly, this method preserves softness and robustness. Li et al. [26,27] proposed a pre-charged pneumatic (PCP) bending soft actuator. In this design, pressurized gas is charged into the actuator through a check valve, and the internal pressure is regulated by motor-driven control of tendon release or tension on the actuator’s dorsal side, thereby enabling bending and unbending motions. Ren et al. [28] proposed a bidirectional tendon-driven soft actuator with a self-pumping design, termed the soft self-pumping actuator (SSPA). Pressurized gas is similarly charged into the SSPA through a check valve. Tendon control redistributes the pre-charged pressurized gas, thereby assisting bending of the SSPA. In parallel, variable-stiffness mechanisms, including antagonistic actuation and related stiffness-tuning strategies, have been widely investigated to enable robots that remain compliant during interaction yet become stiff for load bearing or manipulation when required [29,30].

However, many existing solutions involve unavoidable trade-offs among system complexity, output performance, controllability, and safety. Some approaches require high voltages or substantial thermal inputs, whereas others introduce rigid components that may compromise overall softness. Therefore, a key open challenge is to develop an actuator that preserves the high compliance of pneumatic artificial muscles while eliminating bulky pumps and valves, and that offers a simple and reliable control interface suitable for integration into practical robotic systems.

In this study, we propose a novel method for actuating a pneumatic artificial muscle (PAM) based on the twisting tube actuation (TTA) principle [31], which eliminates the need for external pumps and valves and is straightforward to control. Specifically, when a fluid-filled PAM (e.g., air or water) is twisted at one end, a helical twist pattern forms near the driven end and propagates along the actuator. The twisted segment contracts, forcing the internal fluid to migrate from the twisted region to the untwisted region. Accordingly, we term the resulting actuator the twisting tube artificial muscle (TTAM). In essence, the TTAM combines the characteristics of TTA [31] and a PAM.

**Figure 1:**
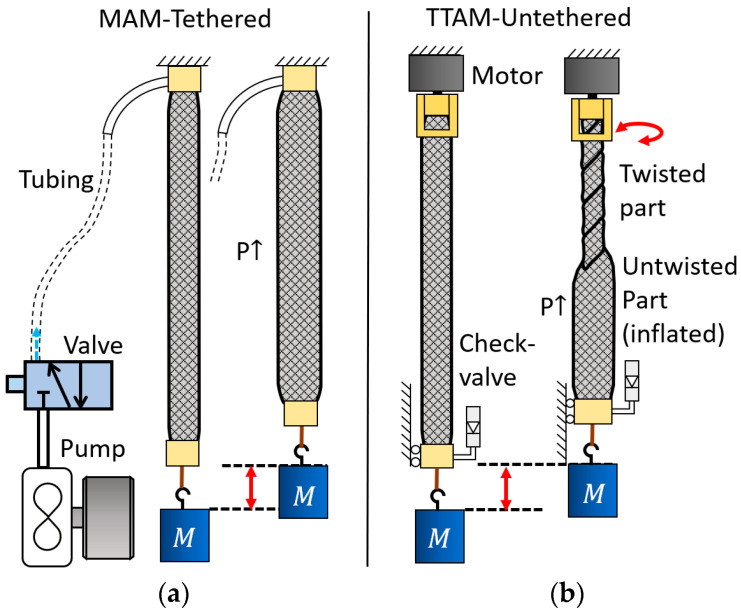
Comparison between two actuation methods. (**a**) A Mckibben artificial muscle (MAM) actuated by pump (*M* means mass block). (**b**) The proposed TTAM showing the contraction of twisted part and untwisted part.

Figure 1 compares a conventional PAM with the proposed TTAM actuator. As shown in Figure 1a, a conventional PAM requires tethering to a pneumatic supply (pump/compressor) and electrically actuated valves to operate.

Figure 1b illustrates the actuation process of the TTAM. The TTAM is pre-charged with pressurized gas at an initial pressure $P_{0}$ and coupled to a motor to provide torsion. When twisted from one end, the fluid in the twisted region is displaced toward the untwisted region. The pressure in the untwisted region consequently increases, causing it to inflate in a manner similar to a conventional PAM. Both the twisted segment and the inflated (untwisted) segment contribute to the overall axial contraction.

For TTAM actuation, external pumps and valves are not required. The TTAM can be driven directly by a motor or by other actuators capable of generating rotary motion. Notably, the working fluid within the TTAM may be a gas, a liquid, or a gas–liquid mixture. When filled with a liquid, the TTAM can produce higher forces because liquids are nearly incompressible. In this paper, we focus on gas-filled TTAMs under different initial pressures.

**Figure 2:**
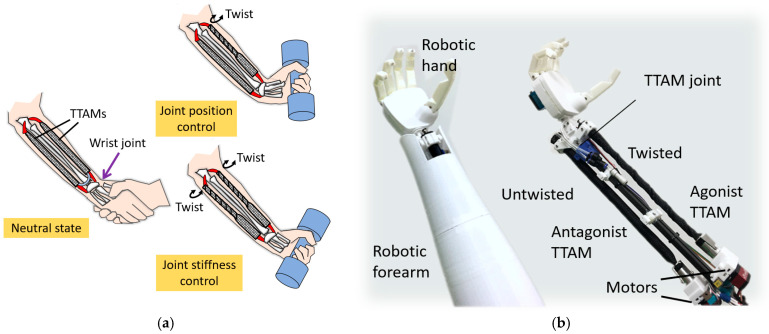
ATTAM wrist joint design (**a**). Concept of the ATTAM wrist drive (**b**). Prototype of an untethered compliant robotic forearm with ATTAM wrist joint.

Antagonistic actuation can be achieved by two or more actuators arranged to act in opposition about a joint, enabling both motion generation and joint stiffness modulation [32,33]. In this study, because the proposed TTAM contracts in a manner analogous to human skeletal muscle, we demonstrate a bioinspired robotic joint driven by an antagonistic pair of TTAMs. The design concept is shown in Figure 2a, in which the joint is configured as a wrist drive for a human forearm. Joint angle and stiffness can be regulated by controlling the twisting angles of the agonist and antagonist TTAMs. In a low-stiffness state, the joint can facilitate safe human–robot interaction; in a high-stiffness state, it can support and manipulate heavier loads. An antagonistic robotic wrist joint prototype based on TTAMs is developed, as shown in Figure 2b.

This paper is organized as follows. Section 2 presents the modeling of the proposed TTAM actuator. Section 3 describes the experimental evaluation of the TTAM. Section 4 presents the design and experiments of a TTAM-driven robotic joint. Section 5 introduces a prototype anthropomorphic robotic forearm. Section 6 concludes the paper and discusses limitations and future work.

## 2. Modeling of the TTAM

**Figure 3:**
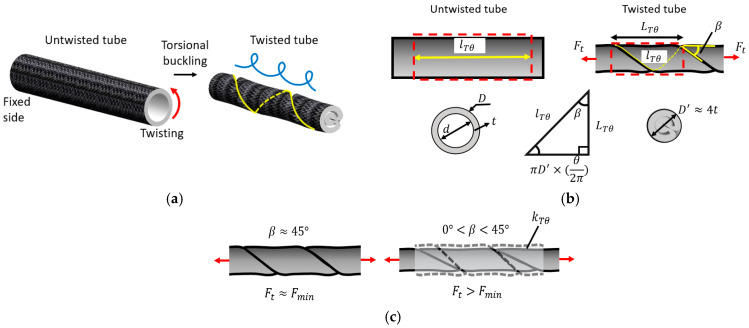
Modeling of twisting tube actuator (TTA). (**a**) Torsional buckling of a tube when being twisted. (**b**) Modeling of TTA for θ twisting angle. (**c**) Schematic diagram of TTA deformation variation with $F_{t}$ and β.

Since TTAM is derived from the twisting tube actuation (TTA) principle, we first summarize the validated TTA model from prior work (Figure 3) [31] as the basis for formulating the twisted segment. The TTAM model is constructed modularly. In the twisted state, the actuator is decomposed into a twisted segment that behaves as a TTA-like element and an untwisted, pressurized segment that behaves as a PAM-like element; these segments are connected in series along the axial direction. This decomposition captures the dominant TTAM physics—geometry-induced contraction in the twisted region and pressure-induced contraction in the untwisted region—while maintaining model tractability.

Unless otherwise stated, we adopt the following assumptions: (i) the working gas is sealed, and leakage during twisting is negligible; (ii) the pressure–volume relation during twisting is approximated as isothermal, i.e., PV≈const. (ideal-gas approximation); and (iii) the TTA geometric relations are derived under the experimentally supported reference conditions $F_{t}\approx F_{min}$ and $\beta \approx 45^{\circ }$ [31], while deviations under higher tension are captured using an effective stiffness term.

As shown in Figure 3a, when an elastic thin-walled tube is continuously twisted (in this study, the tube is constrained by a braided nylon mesh sleeve, i.e., a PAM), a helical torsional-buckling pattern forms along the tube. This pattern expels the internal fluid from the twisted region and induces axial contraction of the tube.

The TTA geometry for a given twisting angle θ is illustrated in Figure 3b. The length of the untwisted tube is denoted by $l_{T\theta }$, the outer and inner diameters by D and d, respectively, and the tube wall thickness by t. When the tube is twisted by an angle θ, the twisted length is denoted by $L_{T\theta }$, and the diameter of the twisted segment is denoted by $D^{\prime }$ (with $D^{\prime }\approx 4t$). Here, β is the lead angle of the helical pattern.

A tensile force $F_{t}$ is required to prevent the twisted tube from knotting or unraveling [31]. Here, $F_{min}$ denotes the minimum tensile force and is estimated experimentally in the following section. As shown in Figure 3c, experimental results in [31] indicate that when $F_{t}\approx F_{min}$ and $\beta \approx 45^{\circ }$, the twisted tube achieves its maximum contraction. When $F_{t}>F_{min}$, the lead angle decreases $\left(0^{\circ }<\beta <45^{\circ }\right)$, and the twisted tube undergoes additional axial stretching. During this process, the tube wall thickness t may also vary, making β difficult to determine accurately. Therefore, we introduce an effective stiffness $k_{T\theta }$ for the twisted segment. When $F_{t}>F_{min}$, the tube is assumed to be twisted under $F_{min}$ and then stretched by the additional force $F_{t}-F_{min}$. Under this framework, the twisted-segment geometry is formulated using the reference condition $F_{t}\approx F_{min}$ and $\beta \approx 45^{\circ }$.

Based on the relationship among the helical path, the helix lead, and the lead angle β [34], the geometric relations between the twisted and untwisted configurations can be derived from the triangle shown at the bottom of Figure 3b. Specifically, the side opposite β (with $\beta \approx 45^{\circ }$) has length $\left(\frac{\theta }{2\pi }\right)\pi D^{\prime }$. Therefore, for a given twisting angle θ, we obtain

(1) $$l_{T\theta }=(\theta /2\pi )\times \pi D^{\prime }/\sin\beta =2\sqrt{2}\theta t,$$

(2) $$L_{T\theta }=(\theta /2\pi )\times \pi D^{\prime }/\tan\beta =2\theta t,$$

The corresponding length change $\Delta L_{T\theta }$ and volume change $\Delta V_{T\theta }$ can be derived from Equations (1) and (2):

(3) $${∆L}_{T\theta }=l_{T\theta }-L_{T\theta }-\frac{F_{t}-F_{min}}{k_{T\theta }}=2(\sqrt{2}-1)\theta t-(F_{t}-F_{min})/k_{T\theta },$$

(4) $${∆V}_{T\theta }=\pi {\left(d/2\right)}^{2}l_{T\theta }=2\sqrt{2}\theta t\pi {\left(d/2\right)}^{2}$$

**Figure 4:**
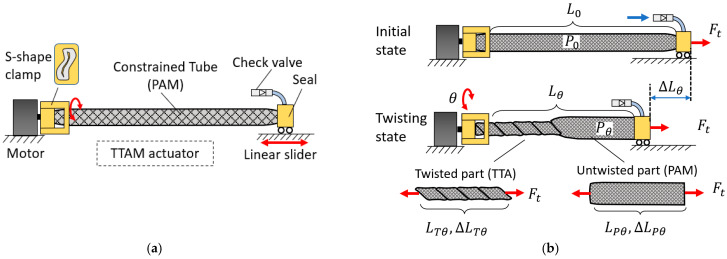
Design and modeling of the TTAM. (**a**) Mechanical design sketch of a TTAM. (**b**) The modelling process of a TTAM.

Design and modeling of the TTAM is presented in Figure 4. A conceptual design of a TTAM is presented in Figure 4a. A constrained tube (i.e., a PAM) is coupled to a motor at one end to provide twisting. An S-shaped clamp is used to introduce a controlled pre-buckling, ensuring that twisting initiates from the motor side [31]. The other end is sealed and equipped with a valve for filling. This end is mounted on a linear slider that constrains rotation and permits only axial translation of the TTAM.

Figure 4b outlines the modeling procedure for the TTAM. In the initial state, the TTAM is inflated to an initial gauge pressure $P_{0}$ (i.e., $P_{0}\geq 0$), has length $L_{0}$, and is subjected to a tensile load $F_{t}$. In the twisted state (Figure 4b), the TTAM is twisted by an angle θ. The corresponding length and axial stiffness are denoted by $L_{\theta }$ and $k_{\theta }$, and the contraction is denoted by $\Delta L_{\theta }$. We model the TTAM as a nonlinear spring with θ-dependent effective length and stiffness, consistent with prior spring-based models for PAMs [35,36] and TSAs [37,38]. Our goal is to derive the relationships among θ, $\Delta L_{\theta }$, and $k_{\theta }$.

As shown in Figure 4b, the TTAM in the twisted state is decomposed into a twisted segment and an untwisted segment. The twisted segment is modeled as a twisting tube actuator (TTA) [31,39,40], whereas the untwisted segment is modeled as a PAM. The lengths and contractions of the twisted (TTA) and untwisted (PAM) segments are denoted by $\left(L_{T\theta },\;\Delta L_{T\theta }\right)$ and $\left(L_{P\theta },\;\Delta L_{P\theta }\right)$, respectively. Therefore, the TTAM length in the twisted state is $L_{\theta }=L_{T\theta }+L_{P\theta }$, and the overall contraction is $\Delta L_{\theta }=\Delta L_{T\theta }+\Delta L_{P\theta }$.

The twisted-segment model for a given twisting angle θ is provided in Equations (1)–(4).

The untwisted segment is modeled using established PAM formulations [15,16]. We estimate the internal gauge pressure $P_{\theta }$ after twisting by an angle θ. Under the isothermal ideal-gas assumption [41], the product of absolute pressure and volume remains constant during twisting, yielding

(5) $$(P_{0}+P_{atm})V_{0}=(P_{\theta }+P_{atm})(V_{0}-\eta {∆V}_{T\theta }),$$

where $V_{0}$ is the initial internal volume of the TTAM before twisting, which can be calculated from the dimensions of the TTAM; that is, $V_{0}=L_{0}{\left(d/2\right)}^{2}\pi$, where $L_{0}$ and d are the length and inner diameter of the TTAM. ${∆V}_{T\theta }$ can be obtained from (4), and η≈0.75~0.8 is the volume correction factor based on experimental test [26].

Therefore, substitute $V_{0}$ and ${∆V}_{T\theta }$ into (5), and we have

(6) $$P_{\theta }=\frac{(P_{0}+P_{atm})L_{0}}{L_{0}-\eta 2\sqrt{2}\theta t\pi }-P_{atm},$$

Once the pressure $P_{\theta }$ inside the TTAM is obtained, the contraction length of the untwisted part (PAM) ${∆L}_{P\theta }$ can be calculated based on the internal pressure change $P_{\theta }$ and length of the untwisted part ($L_{0}-l_{T\theta }$). Therefore, we have

(7) $${∆L}_{P\theta }=\varepsilon_{P_{\theta }}\left(L_{0}-l_{T\theta }\right)=\varepsilon_{P_{\theta }}(L_{0}-2\sqrt{2}\theta t),$$

Here, $\varepsilon (P_{\theta })$ is the PAM contraction ratio as a function of $P_{\theta }$. Although PAM modeling has been studied extensively [15,16], it remains a complex process involving many design-dependent parameters. Moreover, detailed PAM modeling is not the focus of this paper. The relationship between ε and pressure is obtained via experimental fitting in Section 3.

The effective stiffness $k_{\theta }$ of the twisted TTAM is computed by modeling the twisted and untwisted segments as two elastic elements connected in series:

(8) $$k_{\theta }=\frac{k_{T\theta }k_{P\theta }}{k_{T\theta }+k_{P\theta }},$$

Here, $k_{T\theta }$ is the stiffness of the twisted segment. Because $k_{T\theta }$ depends on tube material properties, geometry, and the mesh constraint, it is difficult to model analytically; thus, it is identified experimentally in Section 3. $k_{P\theta }$ denotes the stiffness of the untwisted segment and can be approximated by the stiffness of a PAM. When pressurized, the PAM can be modeled as an air spring. Following [42], its stiffness can be expressed as $k_{P\theta }=\frac{3P_{\theta }L_{P_{\theta }}}{2\pi n^{2}}$, where n is the number of turns for a single thread of the braided mesh tube, n can be expressed by $n=L_{P_{\theta }}/{∆L}_{n}=(L_{0}-2\sqrt{2}\theta t)/{∆L}_{n}$, where ${∆L}_{n}$ is the length of the PAM for one turn of a single thread, which is decided by the parameters of braided mesh tube. Therefore, we have $k_{P\theta }=\frac{3P_{\theta }({{∆L}_{n})}^{2}}{2\pi (L_{0}-2\sqrt{2}\theta t)}$. The stiffness $k_{\theta }$ in (8) can be rewritten as

(9) $$k_{\theta }=\frac{k_{T\theta }(\frac{3P_{\theta }({{∆L}_{n})}^{2}}{2\pi (L_{0}-2\sqrt{2}\theta t)})}{k_{T\theta }+\frac{3P_{\theta }({{∆L}_{n})}^{2}}{2\pi (L_{0}-2\sqrt{2}\theta t)}},$$

## 3. Experimental Characterization of the TTAM

**Figure 5:**
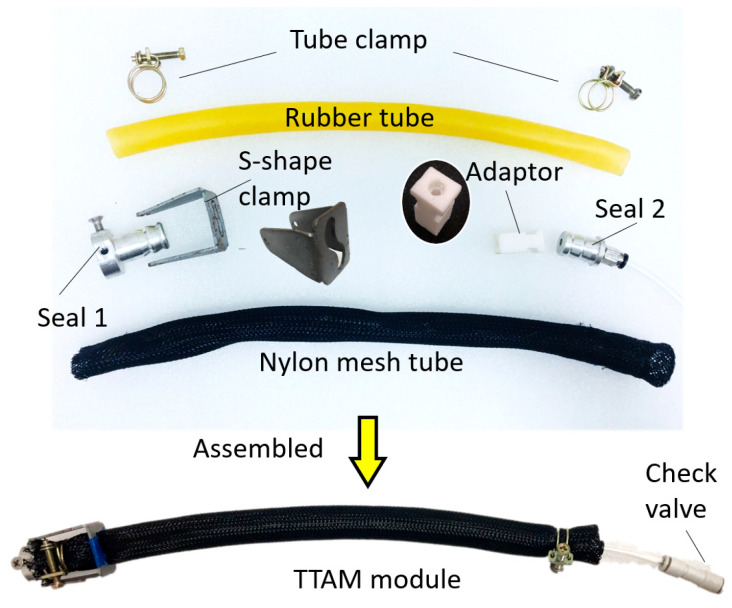
Assembling a TTAM module prototype.

A sample TTAM is manufactured for experimental characterization. The TTAM prototypes are low cost and easy to make, as shown in Figure 5, and major components of a TTAM include a rubber tube (natural rubber tube, outer D = 17 mm, inner d = 14 mm), a braided nylon mesh tube, two tube clamps, two seals (one for motor connection, another for tube connection), an S-shape clamp for pre-buckling of the tube, and an adaptor to help fix the TTAM on the linear guide. All the above-mentioned components are low cost and can be mass-produced. When all the components are ready, it only takes a few minutes to assemble a TTAM.

It is worth noting that through experimental tests, compared with applying only single layer of the braided nylon mesh tube, two layers of braided nylon mesh tube constraints can significantly elongate the service time of the TTAM, while at the same time, does not increase too much of the torsional resistance. In the future, we will try to develop TTAMs that are made of elastic tubes with embedded constraints to further prolong the service life and enhance performance.

**Figure 6:**
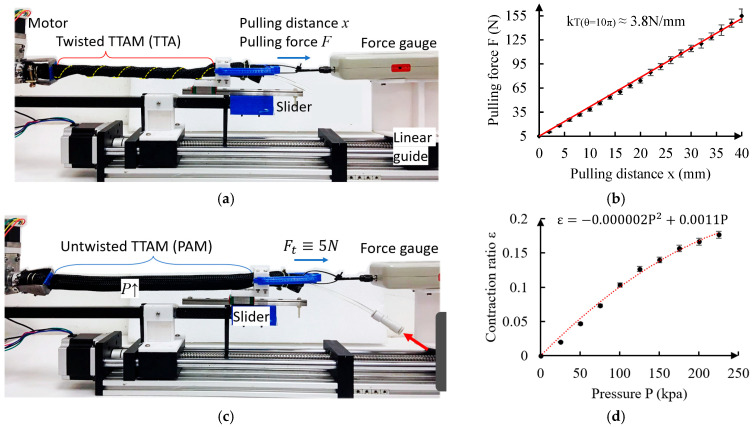
Experimental characterization of stiffness of twisted TTAM (TTA) and contraction ratio of untwisted TTAM (PAM). (**a**) Measurement of the stiffness of the twisted TTAM. (**b**) Experiment results of the stiffness of twisted TTAM. (**c**) Measurement of the contraction ratio of the untwisted TTAM with different internal pressures. (**d**) Experiment results of the contraction ratio vs. the pressure.

As shown in Figure 6, we first conduct two groups of tests to measure the stiffness $k_{T\theta }$ of the twisted TTAM (TTA) and the untwisted TTAM (PAM).

The test platform of the $k_{T\theta }$ is shown in Figure 6a. One end of the TTAM was fixed on the motor, and another end was fixed on a slider. When measuring the stiffness of twisted TTAM, the TTAM was completely twisted for θ=10π. A force gauge was installed on a linear guide that can move back and forth; the force gauge is connected with the right side of the twisted TTAM. A minimum tension force (5 N) to keep the twisted pattern is applied to the twisted TTAM first. The twisted TTAM was pulled and elongated for certain distance (e.g., 40 mm), the test was conducted five times, and the pulling force is measured down for every 2 mm pulling of the twisted TTAM. Relationships of the pulling force F vs. pulling distance x are plotted in Figure 6b based on the average test values (including the error bars). The stiffness of the twisted TTAM (θ=10π) TTA can be calculated from the slope of the fitted line, which is $k_{T10\pi }\approx 3.8\;N/mm$. As a continuous body, the stress inside the twisted TTAM equals everywhere; therefore, based on the Hooke’s law, the stiffness of the twisted TTAM for twisting angle θ can be expressed as $k_{T\theta }\approx \left(\frac{10\pi }{\theta }\right)\times 3.8\;N/mm=\frac{38\pi }{\theta }\;N/mm$.

Figure 6c shows the test of measuring the relationship between contraction ratio ε vs. the inner pressure of the untwisted TTAM (PAM). In the measuring process, we keep the tension force $F_{0}$ as 5 N (minimum pre-tension force of TTA), then we increase the gauge pressure of the untwisted TTAM up to 250 kpa. The contraction ratio of the untwisted TTAM is measured down for every 25 kpa increasing of the pressure. The experiment is conducted 5 times; average values of the experimental results are plotted in Figure 6d. Based on the experimental results, the contraction ratio ε can be expressed as a function of the gauge pressure P:

(10) $$\varepsilon \left(P\right)=-0.000002P^{2}+0.0011P,$$

**Figure 7:**
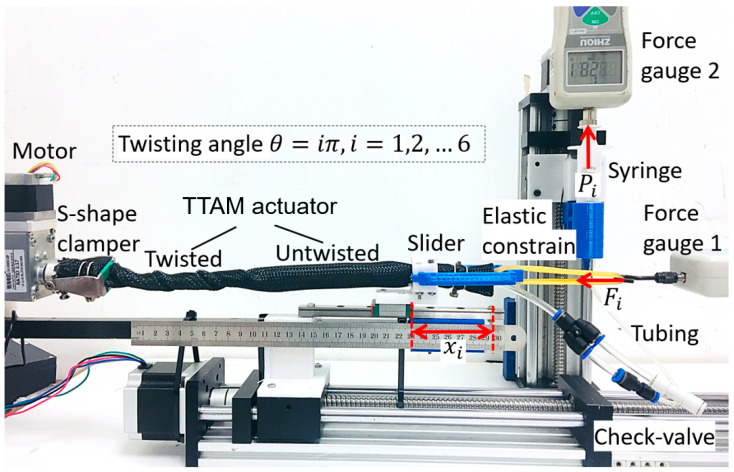
Experiment platform of the TTAM, input variable: twisting angle of the TTAM, output variables: pressure change, contraction, stiffness.

Based on the test platform in Figure 6, a test platform with some added components was built to experimentally characterize the TTAM, as shown in Figure 7. The tubes are connected to right end of the TTAM through a three-way air fitting, one tube is connected with the TTAM, other two-ways tubes are connected with a check-valve (SMC AKH04-00 check valve, SMC Corporation, Tokyo, Japan, cracking pressure: 5 kPa, backpressure: 1000 kPa) and a syringe blocked by force gauge 2.

Three relationships are measured in this test: 1. To measure the relationship between the twisting angle θ and the contraction distance ${∆L}_{\theta }$, the tension force $F_{t}$ that was loaded to the TTAM remained constant; 2. To measure the relationship between the twisting angle θ and the contraction force ${∆L}_{\theta }$, the contraction distance ${∆L}_{\theta }$ of the TTAM remained constant. For both tests, different initial pressures are charged inside the TTAM.

For the TTAM prototype in this experiment, parameters are as follows: length $L_{R}$ of the TTAM in the rest state is 300 mm, the outer diameter of the TTAM D is 23 mm, wall thickness t of the TTAM (include two layers of braided nylon mesh tube) is about 3.5 mm, and length of the TTAM for one turn of a single thread ${∆L}_{n}$ is 90 mm.

**Figure 8:**
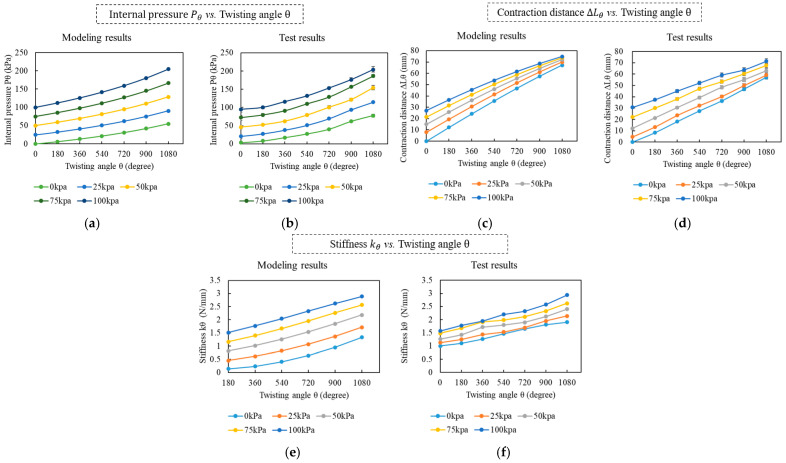
Modeling and experimental results of the TTAM characteristics at different initial pressures. (**a**) Modeling results of the TTAM internal pressure $P_{\theta }$ vs. twisting angle θ. (**b**) Experimental results of the TTAM internal pressure $P_{\theta }$ vs. twisting angle θ. (**c**) Modeling results of the TTAM contraction distance ${∆L}_{\theta }$ vs. twisting angle θ. (**d**) Experimental results of the TTAM contraction distance ${∆L}_{\theta }$ vs. twisting angle θ. (**e**) Modeling results of the TTAM stiffness $k_{\theta }$ vs. twisting angle θ. (**f**) Experimental results of the TTAM stiffness $k_{\theta }$ vs. twisting angle θ.

During the test, the TTAM is charged with five different initially pressures $P_{0}$: 0 kpa, 25 kpa, 50 kpa, 75 kpa, and 100 kpa. There are five groups of tests in total, the TTAM is twisted for θ=6π in every group of tests, and each test group is conducted repeatedly, five times, to eliminate measuring errors. We measure down the internal pressure $P_{\theta }$ and contraction distance ${∆L}_{\theta }$ for every θ=π twisting. To measure the relationship between the twisting angle θ and the internal pressure, the tension force of the TTAM was set as a constant value 5 N. The average values of the experimental results and corresponding modeling results are plotted in Figure 8.

The experimental tests were conducted as follows: 1. The TTAM is charged with different initial pressure ($P_{0}$). When the TTAM was twisted for certain angle θ, the contraction distance ${∆L}_{\theta }$ was measured by a ruler, the tension force $F_{t}$ of the TTAM was measured from the force gauge 1, and the pressure $P_{\theta }$ inside the TTAM was measured and calculated from force gauge 2.

Figure 8a shows the theoretical modeling results of the internal pressure $P_{\theta }$ vs. the twisting angle θ calculated based on (6). Figure 8b shows the experimental test results. The modelling results and the experimental results change with the same trend, the pressure inside the TTAM can be regulated by merely twisting. The larger the initial pressure is, the more significant the pressure change will be. The difference between the modeling and experimental values is relatively small, and the difference can be caused by the measuring errors. Here, we mainly analyze the experimental results. Based on the experimental results, for different initial internal pressures (0 kPa, 25 kPa, 50 kPa, 75 kPa, and 100 kPa), the internal pressure after 6π twisting angles can be up to 74 kPa, 114 kPa, 147 kPa, 182 kPa, and 219 kPa, respectively, with significant increasing (up to 119 kPa).

Figure 8c shows the theoretical modeling results of the relationship of the contraction distance $x_{i}$ vs. the twisting angle iπ calculated based on (9). Figure 8d shows the experimental test results. It is noted that when the initial internal pressure of the TTAM is small, e.g., 0 kPa and 25 kPa, especially 0 kPa, the growing trend of the contraction distance curve in the experimental tests are different from the modeling results. For the experimental results, the slope of the curves approximates a fixed value. For the modeling results, the slope of modeling curve is gradually increasing. The reason is as follows: based on (8) and (9), when the internal pressure is small, the calculated stiffness of the twisted TTAM $k_{i}$ is smaller than the elastic constrain stiffness k=0.6 N/mm; as a result, the contraction distance increase gradually until the internal pressure increased to a certain value. In the modeling of the stiffness of TTAM, for the untwisted part, the stiffness of the tube and constrain is not included in the modeling, and we notice that even at 0 kPa, the TTAM already has some stiffness induced by the material and friction forces, which makes the initial stiffness larger than k=0.6 N/mm in the experimental test.

When the initial internal pressure is increased, i.e., larger than 50 kPa, in the beginning, stiffness of the TTAM will be larger than 0.5 N/mm, and the modeling results basically conform to the experimental results well. For the contraction distance, we analyze the experimental test data. Based on Figure 8d, for different initial internal pressures (0 kPa to 100 kPa), after 6π, compared with the 0 kPa with no twisting, the largest contraction distances ranges from 50 mm to 62 mm, and the contraction ratio of the TTAM after 6π twisting ranges from 16.7% to 20.7%.

Figure 8e shows the theoretical modeling results of the relationship between contraction force $F_{i}$ and the twisting angle iπ calculated based on (9). Figure 8f shows the experimental test results. For the modeling results, it is calculated based on the contraction distance modeling. Therefore, there exists a relatively larger difference when the initial pressure equals to 0 kPa and 25 kPa; the reason has been discussed in the contraction distance part. For the contraction force, we mainly analyze the experimental test data. Based on Figure 8f, for different initial internal pressures (0 kPa to 100 kPa), after 6π, the contraction force ranges in 30 N to 36 N, which means the twisted TTAM can lift 3 kg to 3.6 kg weight with 16.7% to 20.7% contraction.

For the same initial pressure, the contraction force and contraction distance of the TTAM increase with the increasing of the twisting angle; for the same twisting angle of the TTAM, the contraction force and contraction distance increase with the increasing of the initial pressure.

**Figure 9:**
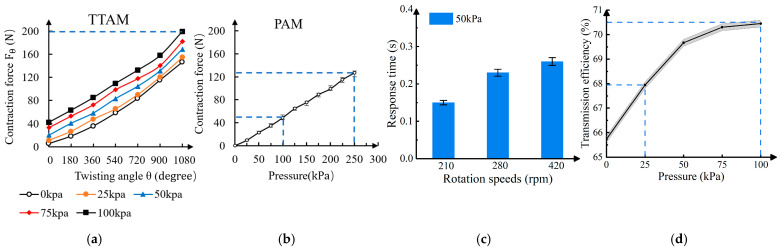
Experimental characterization of the TTAM and comparison with a conventional PAM. (**a**) Experimental results of the TTAM contraction force $F_{\theta }$ vs. twisting angle θ. (**b**) Experimental results of the conventional PAM contraction force $F_{\theta }$ vs. pressure. (**c**) TTAM response time under different twisting speeds (210, 280, and 420 rpm). (**d**) Transmission efficiency of the TTAM under different pressures.

To measure the maximum contraction force generated by the TTAM during twisting under different initial pressures, we repeated the experiment in Figure 7 while replacing the elastic constraint with an inextensible nylon ribbon. Under this condition, the TTAM exhibits negligible axial shortening. The TTAM was twisted to 6π under the same set of initial internal pressures as in the previous experiment. For each pressure level, five trials were performed to reduce experimental variability. The results are shown in Figure 9a. The contraction force increases with initial pressure, and the peak force ranges from 130 to 200 N as the initial pressure increases from 0 to 100 kPa with only 6π twisting, indicating that the TTAM is suitable for heavy-load applications such as robotic limbs. The measured force is substantially higher than that of our previously reported twisting tube actuator using a polyester mesh constraint [31], and it also exceeds the force produced by a parallel TTA configuration, which reached 120 N with water as the working fluid. The TTAM prototype weighs approximately 121 g, corresponding to a load-to-weight ratio of 200 N/1.21 N≈165. When the motor mass is included (62 g in the final robotic-arm prototype), the load-to-weight ratio is approximately 200 N/1.83 N≈108.

For comparison, we also measured the contraction force of a conventional PAM under different pressures. The PAM used in this test was identical to the TTAM actuator body but was not twisted; instead, it was directly pressurized by an external gas supply. The pressure was increased from 0 to 250 kPa. The upper limit of 250 kPa was selected because the internal pressure of the TTAM during twisting did not exceed this value. Contraction force was measured in 25 kPa increments, with five trials at each pressure level. The results are plotted in Figure 9b. The PAM produces substantially lower contraction forces than the TTAM. With an initial pressure of 0 kPa, the TTAM reaches a peak force of 146 N after 6π twisting; with an initial pressure of 100 kPa, the peak force approaches 200 N. In contrast, the PAM produces only 48 N at 100 kPa, and even at 250 kPa, the force is approximately 126 N, which remains lower than the TTAM force at 0 kPa after 6π twisting. These results indicate that the TTAM can generate substantially higher contraction forces than a conventional PAM under comparable pressure levels.

To evaluate the response speed of the TTAM system, we measured the response time at three rotational speeds of 420, 280, and 210 rpm. For each speed, the tube was twisted for seven turns, and five repeated trials were conducted. The results are summarized in Figure 9c, where each bar represents the time required for the end pulling force to rise to its maximum value. The plotted values are the averages over the five trials, and the error bars indicate the upper and lower bounds of the variation across trials. To quantify the system response delay, we first measured the motor rotation time required to complete seven turns at each speed, which corresponds to the ideal case in which motor rotation is transmitted instantaneously. We then measured the actual time required for the TTAM to contract and for the end pulling force to reach its peak. The difference between the measured peak-force time and the corresponding motor rotation time is defined as the response delay reported in Figure 9c. The experimental results show a response delay ranging from 0.15 to 0.26 s for both pressure and pulling force across the three rotational speeds. This delay is primarily attributed to the elasticity of the rubber tube, which slows torque transmission relative to a rigid shaft. Moreover, the response delay depends on rotational speed: it is approximately 0.15 s at 210 rpm and increases to about 0.26 s at 420 rpm.

We conducted tests under varying air pressure conditions to evaluate the transmission efficiency of the TTAM. The transmission efficiency of the system was defined as the ratio of the motor input force to the terminal contraction force of the TTAM, both of which were recorded during the experiment. In the experiment, the tube was twisted for three turns under varying air pressure conditions, with five repeated trials conducted at the same rotational speed. The results are shown in Figure 9d. The results show that the transmission efficiency of the system increases with pressure: it reaches 70.4% at 100 kPa, approximately 8% higher than at 0 kPa (65.3%). This increase is primarily due to the enhanced stiffness response of the system resulting from higher pressure.

Based on the modeling results and experimental characterization, several features of the TTAM can be concluded as follows:

1. TTAM actuators can generate contraction during twisting, just like a MAM. Connection with pump and electrical valves through tubes are eliminated for TTAM. Once charged with initial pressures, contraction of the TTAM can be controlled by controlling the twisting angles of the motor without connecting to external air sources.
2. By charging different initial pressures, the contraction distance and contract force of the TTAM will change accordingly. Based on the experimental results, the larger the initial pressure is, the larger the contraction distance and contraction force will be.

## 4. Design and Characterization of an Antagonistic Robotic Joint Driven by TTAMs

### 4.1. Design of the Antagonistic Robotic Joint

Position control and variable stiffness control can be achieved through the antagonistic design [31], which can always be found in compliant robotic arms design with PAMs [18,35,43]. The TTAM actuator is compliant and generates contractile force just like PAMs; therefore, two TTAMs can be installed in an antagonistic way to drive a robotic joint, named as Antagonistic TTAM (ATTAM) joint.

**Figure 10:**
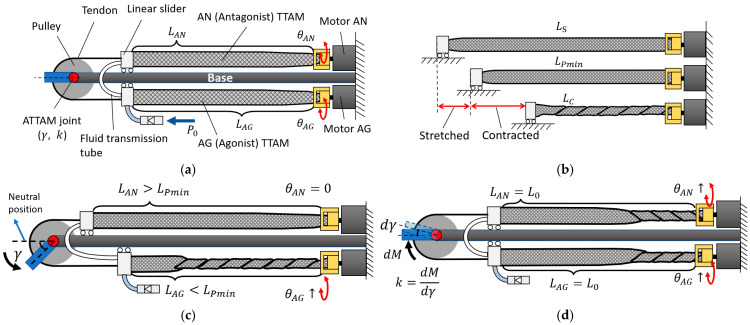
Antagonistic robotic joint with TTAMs. (**a**) Mechanical design of the ATTAM joint. (**b**) Different states of TTAM during actuation. (**c**) Position control of the ATTAM joint. (**d**) Stiffness control of the ATTAM joint.

The design and working principle of the ATTAM joint is shown in Figure 10. In Figure 10a, a pair of TTAMs are mounted on a base; one is defined as Agonist (AG) TTAM, the other is Antagonist (AN) TTAM. The AG and AN TTAMs are connected through a fluid transmission tube. Therefore, during the actuation process, the pressure inside the AG and AN TTAMs remains the same all the time. Free ends of the AG and AN TTAMs are fixed on two linear sliders that can move back and forth along the base; a pulley is driven by the AG and AN TTAMs through tendons. We define $\theta_{AG}$ and $\theta_{AN}$ as the twisting angle of the AG TTAM and AN TTAM, respectively, and γ and k are the ATTAM joint angle and joint stiffness.

In this design, the AG and AN TTAMs are charged with an initial pressure $P_{min}$, so that they could have some elasticity before any twisting. We define the length of the TTAM with $P_{min}$ as $L_{P_{min}}$. It can be stretched to $L_{S}$, and it contracts to $L_{C}$ when it is completely twisted, as shown in Figure 10b.

As the AG and AN TTAM are connected through the air tube, the pressure inside them remains the same all the time; therefore, based on the modeling and test results in Figure 8, the contraction length and stiffness of the AG and AN TTAM are positively correlated with their twisting angles.

The ATTAM joint angle γ is the pulley’s rotation angle from the neutral position. When $\theta_{AG}=\theta_{AN}$, the contraction length ${∆L}_{\theta AG}={∆L}_{\theta AN}$, and the stiffness $k_{\theta AG}=k_{\theta AN}$, the ATTAM joint will stay at the neutral position; when $\theta_{AG}>\theta_{AN}$, the contraction length ${∆L}_{\theta AG}>{∆L}_{\theta AN}$, and the stiffness $k_{\theta AG}>k_{\theta AN}$; as a result, the ATTAM joint will rotate towards the AG TTAM direction to balance the force; vice versa, when $\theta_{AG}<\theta_{AN}$, the joint will rotate towards the AN direction.

The ATTAM joint stiffness is defined as k=dM/dγ, which is the unit torque dM required to rotate the joint for unit angle dγ. When both $\theta_{AG}$ and $\theta_{AN}$ increase, the stiffness $k_{\theta AG}$ and $k_{\theta AN}$ will increase, and the AG and AN TTAM will both contract, as a result, the ATTAM joint stiffness will increase; when both $\theta_{AG}$ and $\theta_{AN}$ decrease, the ATTAM joint stiffness will decrease; when $\theta_{AG}$ increases, $\theta_{AN}$ decreases, and the ATTAM joint stiffness will remain a constant value.

### 4.2. Experimental Characterization of the ATTAM Joint

**Figure 11:**
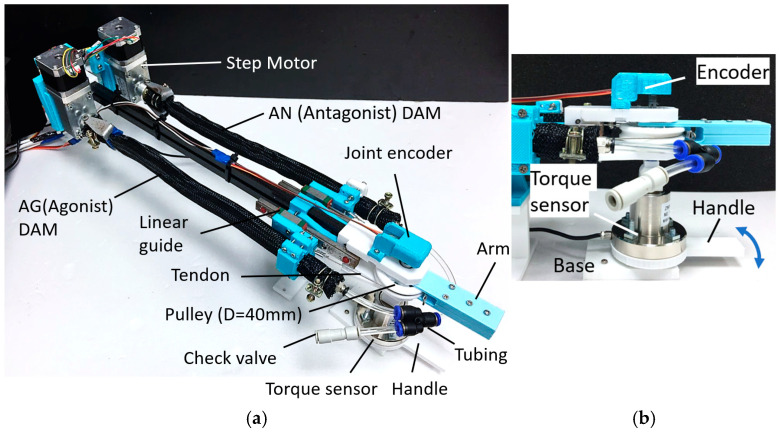
Experimental platform of ATTAM joint. (**a**) Components of the experimental platform. (**b**) Sensors for measuring joint angle and joint stiffness.

An experimental platform is designed and built to characterize the ATTAM joint, as shown in Figure 11. Two TTAMs are installed in an antagonistic way, as shown in Figure 11a. On the motor side, two step motors with worm-gear reducers (maximum torque 3 Nm) are used for actuation, and the rotation angle and rotation direction of the step motors can be controlled.

On the joint side, free ends of the TTAMs are fixed on linear guides, and when twisting the TTAM, the free end will contract and move along the linear guides. The free ends are also connected to a pulley through steel wires; a 40 mm diameter pulley is used as the robotic joint, and a 3D-printed short “arm” is mounted on the pulley to show the joint rotation angle. Two TTAMs are connected through air tubes; a check-valve is connected to the two TTAMs through a three-way tube fitting, and different initial pressure can be charged to the system for testing.

In Figure 11b, a joint encoder is mounted above the joint to measure the rotation angle, and a static torque sensor with a 3D-printed handle is installed below the joint to measure the joint stiffness.

**Figure 12:**
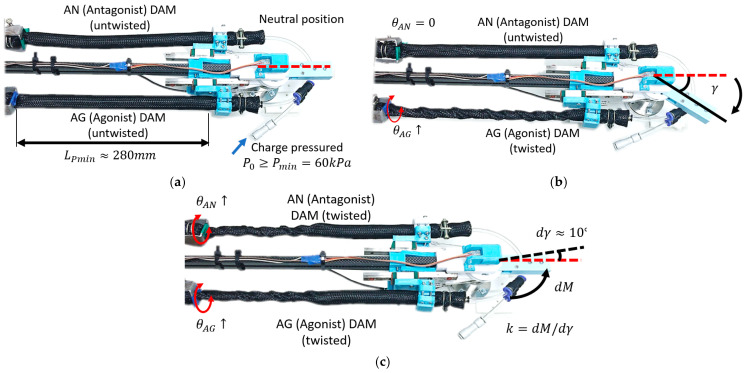
Experimental characterization of the ATTAM joint. (**a**) The neutral position of the ATTAM joint. (**b**) Measurement of the ATTAM joint angle. (**c**) Measurement of the ATTAM joint stiffness.

Figure 12 shows the test process of the ATTAM joint. In Figure 12a, the ATTAM joint is in the neutral position, and the AG and AN TTAMs were charged with certain pressure and in contract state, so that it is stretchable and has natural compliance just like an air spring. The length of the TTAM prototype in the rest state $L_{R}$ is 300 mm, and we define the minimum pressure $P_{min}$ for the ATTAM as 60 kPa. When the TTAM is charged with 60 kPa, the contraction ratio is about 0.06, and the contraction length is about 20 mm. Therefore, the $L_{P_{min}}$ is about 280 mm. In the experimental tests, the ATTAM joint is charged with different pressure levels, i.e., 60 kPa, 80 kPa, and 100 kPa.

As discussed in the modeling process, the ATTAM joint is controlled by controlling the twisting angle of the AG and AN TTAMs, i.e., controlling the i and j. For each set of i and j, the joint angle $\gamma_{i,j}$ and joint stiffness $k_{i,j}$ are different. Therefore, in the experimental characterization process, we will mainly consider the following two cases: 1. for the joint angle γ, one side of the TTAM is twisted, i.e., the AG TTAM; that is, to increase the $\theta_{AG}$, the AN TTAM twisting angle $\theta_{AG}$ remains as zero (Figure 12b); 2. for the joint stiffness k, we remain the joint position fixed, i.e., $\theta_{AG}=\theta_{AN}$, while increasing the twisting angle $\theta_{AG}$ and $\theta_{AN}$ simultaneously (Figure 12c).

**Figure 13:**
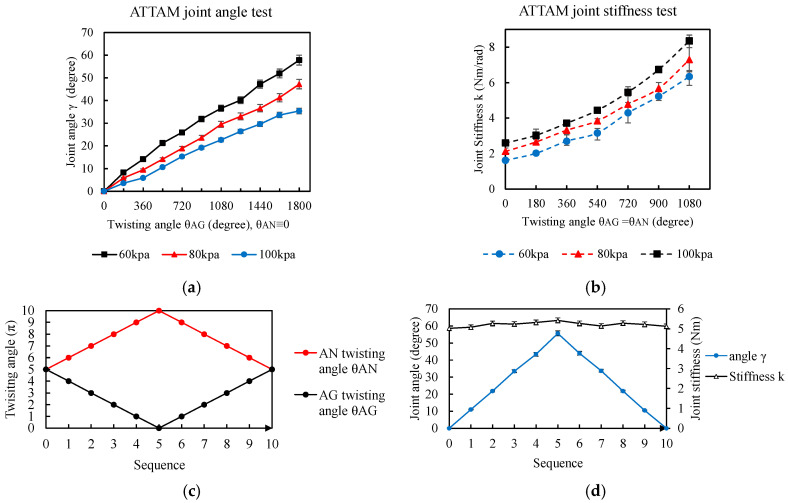
Experimental results of the joint angle and joint stiffness. (**a**) Experimental results of TTAM joint angle γ vs. twisting angle $\theta_{AG}$, $\theta_{AN}\equiv 0$. (**b**) Experimental results of the ATTAM joint stiffness k vs. twisting angle $\theta_{AG}=\theta_{AN}$. (**c**) Control sequence of the twisting angle $\theta_{AG}$ and $\theta_{AN}$. (**d**) Experimental results of the joint angle and joint stiffness change with control sequence of (**c**).

The first test measures the relationship between the joint angle γ and the AG TTAM twisting angle $\theta_{AG}$ (Figure 12b). During the test, the AG TTAM is twisted 1800 degree, and for every 180 degree twisting, the joint angle can be measured from the joint encoder. For different pressure levels (60 kPa, 80 kPa, and 100 kPa), the experiments are conducted five times. Figure 12a shows the modeling results of the joint angle γ vs. the AG TTAM twisting angle $\theta_{AG}$, and Figure 13a shows the test results. Basically, the joint angle witnesses an increase with the increasing of the AG TTAM twisting angle, and the ATTAM joint bends towards the twisted AG TTAM side. For the same twisting angle of AG TTAM, the larger the initial pressure is, the smaller the joint angle will be. When the initial pressure is 60 kPa, after 1800 degree twisting of the AG TTAM, the joint angle reaches about 57 degree, and when the initial pressure increased to 80 kpa, the range of the joint angle is limited in 46 degree, and the joint angle is limited in 34 degree when the initial pressure increased to 100 kPa. The reason for this is that the increased initial pressure will increase the stiffness of the AN TTAM on the other side; therefore, the elongation of the AN TTAM will be smaller, which lead to decreasing of the joint angle.

To characterize the relationship between the ATTAM joint stiffness and twisting angle of AG and AN TTAMs, we also analyze a typical case here, which is the twisting angle of AG and AN TTAMs increasing simultaneously, and $\theta_{AG}$ equals to $\theta_{AN}$ all the time. In this condition, the joint will remain at the neutral position, and the joint stiffness will increase with the increasing of AG and AN twisting angle. As shown in Figure 12c, a test is conducted to measure the relationship between joint stiffness vs. twisting angles with different initial pressures. The experiments are conducted five times for different charged pressures (60 kpa, 80 kpa, and 100 kpa). The AG and AN TTAMs are both twisted for 1080 degrees. To measure the joint stiffness, for every 180-degree twisting, the robotic joint is turned by an external torque dM for dγ≈10°; the measured joint stiffness can be calculated as dM/dγ.

The test results are plotted in Figure 13b. For the same initial pressure, the joint stiffness increases significantly with the increase in $\theta_{AG}$ and $\theta_{AN}$; for the same twisting angle, the larger the initial pressure is, the larger the joint stiffness will be. For different initial pressures, 60 kPa, 80 kPa, and 100 kPa, before any twisting, the ATTAM joint has natural compliance, where the stiffnesses are relatively smaller, about 1.6 Nm/rad, 2.11 Nm/rad, and 2.59 Nm/rad, respectively; after 6π twisting of AG and AN TTAMs, for different initial pressures, the joint stiffness increases up to 6.33 Nm/rad, 7.29 Nm/rad, and 8.34 Nm/rad, with nearly 3.8, 3.45, and 3.22 fold increase in the joint stiffness, respectively. Based on the experimental results, for both before and after twisting, the larger the initial pressure is, the larger the joint stiffness will be; however, it is noted that if only considering the stiffness increment, a smaller initial pressure results in a higher change rate.

Based on the test results shown in Figure 13a,b, it can be concluded that a larger initial pressure can result in a larger joint stiffness of the ATTAM joint; however, the maximum joint angle is smaller. Therefore, for different application requirements, different initial pressure levels should be charged accordingly.

We propose a simple control strategy to actuate the ATTAM joint, to control the joint position at a steady joint stiffness. Based on the modeling and test results of the joint angle, when one TTAM is twisted 1800 degree, the joint reaches the maximum joint angle. We assume the twisting angle of AG and AN TTAMs always satisfy the equation $\theta_{AG}+\theta_{AN}=1800\;degree$. Before actuation, the ATTAM joint stiffness is increased by twisting both AG and AN 900 degree. To control the joint angle of the ATTAM joint (we let the ATTAM joint rotate towards the AG direction), we increase the $\theta_{AG}$ to 1800 degree, and decrease the $\theta_{AN}$ to 0, and the interval of control is a 180 degree twisting angle.

Controlling sequences of the $\theta_{AG}$ and $\theta_{AN}$ are presented in Figure 13c. In the experimental test, the initial pressure is set as 60 kPa, and the test is repeated five times to obtain the average values. The experimental results are presented in Figure 13d, where it can be seen that with the proposed control strategy, the joint angle changes linearly with the change of the twisting angle, and the joint stiffness is broadly stable, with a slight fluctuation during actuation. In the actuation process, the joint angle ranges from 0 to 55 degrees, and the control resolution of the joint angle is about 11 degrees for every 180 degrees twisting angle. The ATTAM joint stiffness remains at a steady level with 0.3 Nm/rad vibration (5.12~5.42 Nm/rad). Therefore, the proposed control strategy can realize linear joint position control at a relatively high and steady joint stiffness.

## 5. A Prototype ATTAM Wrist Joint

**Figure 14:**
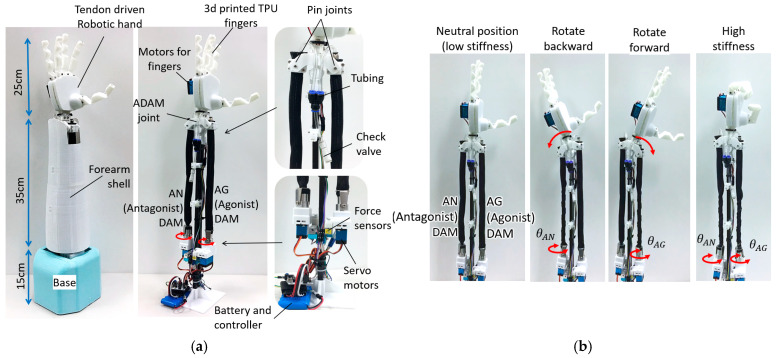
An anthropomorphic robotic forearm with ATTAM wrist joint. (**a**) Mechanical design of the robotic forearm with ATTAM wrist joint. (**b**) ATTAM joint position control and stiffness control.

To demonstrate the ATTAM joint’s practicability, an anthropomorphic robotic forearm prototype is designed and manufactured as shown in Figure 14a. The dimension of the robotic forearm is about 1.2 times of an adult male forearm. A 3D-printed tendon driven robotic hand is installed on the wrist joint for grasping. Compared with the test platform in Figure 11, the robotic wrist joint is designed in a more compact and lightweight manner. Instead of using the linear guide at the free end of each TTAM, the TTAMs are connected with the wrist joint directly through two pin joints. Two small and powerful motors (25 kg·cm torque, and 65 g weight) are used for twisting the AG and AN TTAMs, and the rotation angle and rotation speed of the motors can be controlled. A wireless controller and a 7.5 V battery are mounted on the base. Total weight of the robotic forearm is only about 1.1 kg (weight of the arm is 800 g, the weight of the robotic hand is about 300 g). When AG or AN TTAM is twisted, respectively, the wrist will rotate back and forth, and rotation range of the wrist joint is about ±45°. When AG and AN TTAMs are twisted simultaneously, the wrist joint will remain in the neutral position, while the joint stiffness will increase accordingly.

**Figure 15:**
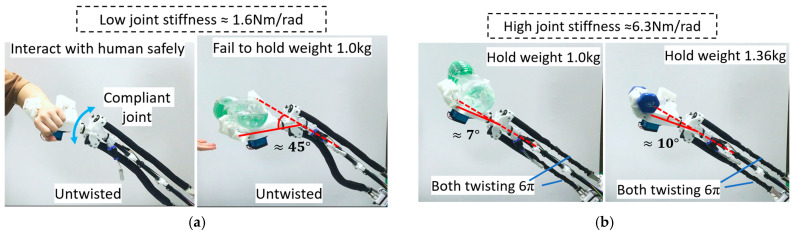
Variable stiffness wrist joint demonstration. (**a**) Low joint stiffness (1.2 Nm/rad) with natural compliance. (**b**) High joint stiffness (6.3 Nm/rad) state can hold heavy objects.

Flexibility and adjustable stiffness are critical factors for robotics development. Natural compliance of a robot joint is essential for human–robot safety interaction [44]. The ATTAM joint has natural compliance as the AG and AN TTAMs can be regarded as two PAMs with adjustable internal pressure. The ATTAM system is charged with 80 kpa pressure in this experiment. When the AG and AN TTAMs are not twisted at all, the joint has a low stiffness (1.6 Nm/rad), while having a high compliance (Figure 15a). In some application scenes such as the rehabilitation training of patient, or interacting with fragile objects, compliance can improve the comfort and safety level, as well as resilience.

However, it is noted that the wrist cannot hold too much weight at a low stiffness state, i.e., the joint deflection angle is about 45° when carrying a large bottle of water (1.0 kg) (Figure 15a). In these cases, where heavy objects are needed to be manipulated, a 3.9 fold stiffness increase can be realized by twisting AG and AN TTAMs simultaneously for 6π without need to connect with any extra gas source, as shown in Figure 15b. The tension force of the ATTAM joint can be measured by the tension sensor at the motor side; when the tension force reaches the target value, the ATTAM is in high stiffness state (joint stiffness 6.3 Nm/rad). In the high stiffness state, the joint can hold the 1.0 kg water bottle with 7° deflection, and when holding a 1.36 kg dumbbell, the deflection angle is about 10°. Demonstration of the anthropomorphic robotic forearm is included in the Video S1.

## 6. Conclusions and Discussion

This study proposes a novel artificial muscle, termed the twisting tube artificial muscle (TTAM). The TTAM is derived from the twisting tube actuation (TTA) principle and integrates key characteristics of pneumatic artificial muscles (PAMs). Unlike conventional PAM-driven systems, the TTAM eliminates the need for external pumps and pressure regulators. A prescribed amount of working fluid is sealed within the actuator, and regulating the twisting angle enables coordinated control of internal pressure, axial contraction, and effective stiffness. Modeling and experiments consistently show that increasing the initial pressure generally increases contraction and output force. In addition, the initial pressure can be adjusted via a check valve to accommodate different task requirements.

To demonstrate practical applicability, we developed a bioinspired antagonistic robotic joint (ATTAM) driven by a pair of TTAMs. Like the TTAM, the ATTAM operates without an external pump and exhibits intrinsic compliance. Joint angle and stiffness are modulated by controlling the twisting angles of the agonist and antagonist TTAMs. Furthermore, when the actuators are charged with a gas–liquid mixture (e.g., water and pressurized gas), the joint stiffness increases substantially in our prototype (from approximately 1.6 to 9.4 N·m/rad), highlighting the potential for compliant robotic systems that require both safe interaction and load-bearing capability.

Despite these encouraging results, several limitations remain and motivate future work. Long-term durability remains an important consideration because repeated twisting may progressively degrade the constraint layer, thereby reducing the repeatability and accuracy of TTAM actuation. Accordingly, future efforts will focus on improving constraint-layer robustness and overall structural reliability. In addition, the influence of working-fluid selection warrants further investigation. While this study focuses on gas-filled TTAMs, alternative working fluids or gas–liquid mixtures may improve efficiency and output performance; their effects on static and dynamic actuator behavior will therefore be evaluated systematically. At the joint level, the current ATTAM prototype represents a single design point; future work will explore alternative mechanical architectures to improve integration, compactness, and performance. Moreover, although sensors have been integrated to measure joint position and stiffness, closed-loop control has not yet been fully developed. In particular, hysteresis may arise from elastomer viscoelasticity, frictional contact within the braided constraint, and pneumatic losses during internal fluid redistribution, and it may degrade control accuracy and repeatability. Because hysteresis was not explicitly quantified in this work, hysteresis modeling and compensation are beyond the scope of the current framework; future research will therefore incorporate hysteresis characterization and develop hysteresis-aware sensing, modeling, and compensation strategies to improve tracking accuracy and repeatability within a closed-loop control framework. Finally, broader application demonstrations will be pursued to validate TTAM/ATTAM in use cases where untethered operation, compliance, and tunable stiffness provide clear advantages.

## Data Availability

Dataset available on request from the authors. The raw data supporting the conclusions of this article will be made available by the authors on request.

## Supplementary Materials

The following supporting information can be downloaded at https://www.mdpi.com/article/10.3390/biomimetics11010038/s1; Video S1: actuation of the ATTAM wrist joint.
